# Clinical feasibility of deep learning-based synthetic CT images from T2-weighted MR images for cervical cancer patients compared to MRCAT

**DOI:** 10.1038/s41598-024-59014-6

**Published:** 2024-04-12

**Authors:** Hojin Kim, Sang Kyun Yoo, Jin Sung Kim, Yong Tae Kim, Jai Wo Lee, Changhwan Kim, Chae-Seon Hong, Ho Lee, Min Cheol Han, Dong Wook Kim, Se Young Kim, Tae Min Kim, Woo Hyoung Kim, Jayoung Kong, Yong Bae Kim

**Affiliations:** Department of Radiation Oncology, Yonsei Cancer Center, Heavy Ion Therapy Research Institute, Yonsei University College of Medicine, 50-1 Yonsei-Ro, Seodaemun-gu, Seoul, 03722 Korea

**Keywords:** Synthetic CT images, MR images, Deep learning, MRCAT, Cervical cancer, Radiotherapy, Biomedical engineering, Magnetic resonance imaging, Radiography, Tomography

## Abstract

This work aims to investigate the clinical feasibility of deep learning-based synthetic CT images for cervix cancer, comparing them to MR for calculating attenuation (MRCAT). Patient cohort with 50 pairs of T2-weighted MR and CT images from cervical cancer patients was split into 40 for training and 10 for testing phases. We conducted deformable image registration and Nyul intensity normalization for MR images to maximize the similarity between MR and CT images as a preprocessing step. The processed images were plugged into a deep learning model, generative adversarial network. To prove clinical feasibility, we assessed the accuracy of synthetic CT images in image similarity using structural similarity (SSIM) and mean-absolute-error (MAE) and dosimetry similarity using gamma passing rate (GPR). Dose calculation was performed on the true and synthetic CT images with a commercial Monte Carlo algorithm. Synthetic CT images generated by deep learning outperformed MRCAT images in image similarity by 1.5% in SSIM, and 18.5 HU in MAE. In dosimetry, the DL-based synthetic CT images achieved 98.71% and 96.39% in the GPR at 1% and 1 mm criterion with 10% and 60% cut-off values of the prescription dose, which were 0.9% and 5.1% greater GPRs over MRCAT images.

## Introduction

Magnetic resonance (MR) imaging enables for highlighting the specific tissues by manipulating pulse-sequences. This ability facilitates the tumor detection and delineation for both diagnostic and therapeutic purposes. Unlike computed tomography (CT), however, MR image does not provide physical information such as electron density for dose calculation. Contrarily, the intensity of CT image, denoted by Hounsfield Unit (HU), represents physical information. By matching the HU and electron (physical) density throughout measurements and/or Monte Carlo simulations, CT images can be employed for dose calculation, followed by treatment planning for radiotherapy (RT). For this reason, MR images have been considered subsidiary in RT. There has been a high demand for making MR images more useful by generating CT-like images, called synthetic CT^1–4^.

The generation of synthetic CT images from MR images is not a new idea, while it has been studied for decades. Multiple approaches have been proposed, including segmentation-based^5–8^ and atlas-based^9–14^. Segmentation-based techniques basically separate multi-echo MR images into different substances: water, fat and bone. Then, the intensities of water and bone substances of MR images are converted to CT numbers with reference to a conversion curve between MR intensity and CT number. This approach could produce synthetic CT images well-aligned to MR images, while the performance relied on parameters such as a priori segmentation, and intensity interpolation. A commercially available system, called MR for calculating attenuation (MRCAT), was developed and released that generates a type of synthetic CT images from MR images with a specific pulse sequence^15^. Atlas-based approaches generate CT images based on deformation information between the given MR image and one of the similar MR images stored in an atlas library. This method mainly depends on the deformation accuracy and similarity of the MR images in atlas library.

A newer concept for synthetic CT image generation is to utilize a learning-based method with the aid of recent breakthroughs of machine learning^16–18^ and deep learning^19–21^ algorithms. The image translation from MR to CT is considered to be a non-linear estimation, which can be modeled by statistical approaches, which involves constructing a network architecture that is designed to be optimized with numerous datasets. This data-driven approach would be able to overcome such drawbacks as the deformation accuracy and imperfect segmentation throughout a thresholding operation that appeared in the existing methods. Previously, the network consists of extracting features from MR images and generating the synthetic CT images based upon the extracted features. The success of this approach depended on reliably finding and matching the features of the given image. To better predict the non-linear model between input and output, convolution kernels were combined with the deep-neural network, which led to convolutional neural networks (CNNs) These networks optimize a number of convolution kernels between 2 and 3D images imported to input and output of the networks^22–27^. Recently, newer generative network architectures such as generative adversarial network (GAN)^28,29^, vision transformer^30–32^, and diffusion probabilistic models^33,34^ have been applied to generating synthetic CT generation from MR images.

This work deals with medical imaging for patients undergoing cervical cancer, which is known as the second most common female malignant tumor. Recent breakthroughs have revolutionized cervical cancer detection based on deep learning^35,36^ and treatment with genome-based^37,38^ and immune system-associated^39^ methodologies. In radiation therapy for cervical cancer, MR image have played a crucial role in delineating target volume. Commercially available segmentation-based synthetic CT generating algorithms, including MRCAT, was initially applied to the pelvis and prostate regions in most cases. Hence, the pelvic region was chosen for this work, which investigates both segmentation-based (MRCAT) and learning-based synthetic CT image generation from MR images.

It was found that many studies have developed deep neural networks capable of generating synthetic CT images from given MR images for several body sites^40–43^, including the pelvis and the cervix^44–46^, which allowed the application of a clinically approved MRCAT pelvis protocol. Only a few, however, investigated the potential use of radiation therapy application by incorporating dosimetry studies, which failed to provide a comprehensive verification procedure to assess the clinical relevance of the generated synthetic CT images. Thus, our main contributions of this work were as follows:

- This work focused on developing a deep learning-based synthetic CT images from (T2-weighted) MR images for cervical cancer patients from well-aligned and pre-process datasets.
- It aimed to demonstrate the clinical relevance of the deep learning-based synthetic CT images for radiotherapy by making comparison to clinically approved MRCAT images from a commercial system.

To achieve our goals, we applied thorough pre-processing to the pairs of T2-weighted MR and CT images for cervical cancer patients that ensures qualified alignment between input and output of the proposed deep neural network. Additionally, we emphasized a verification process for proving clinical feasibility of the deep learning-based synthetic CT images by calculating dose distributions, which were compared against those from planning CT and commercial MRCAT images.

## Results

**Figure 1:**
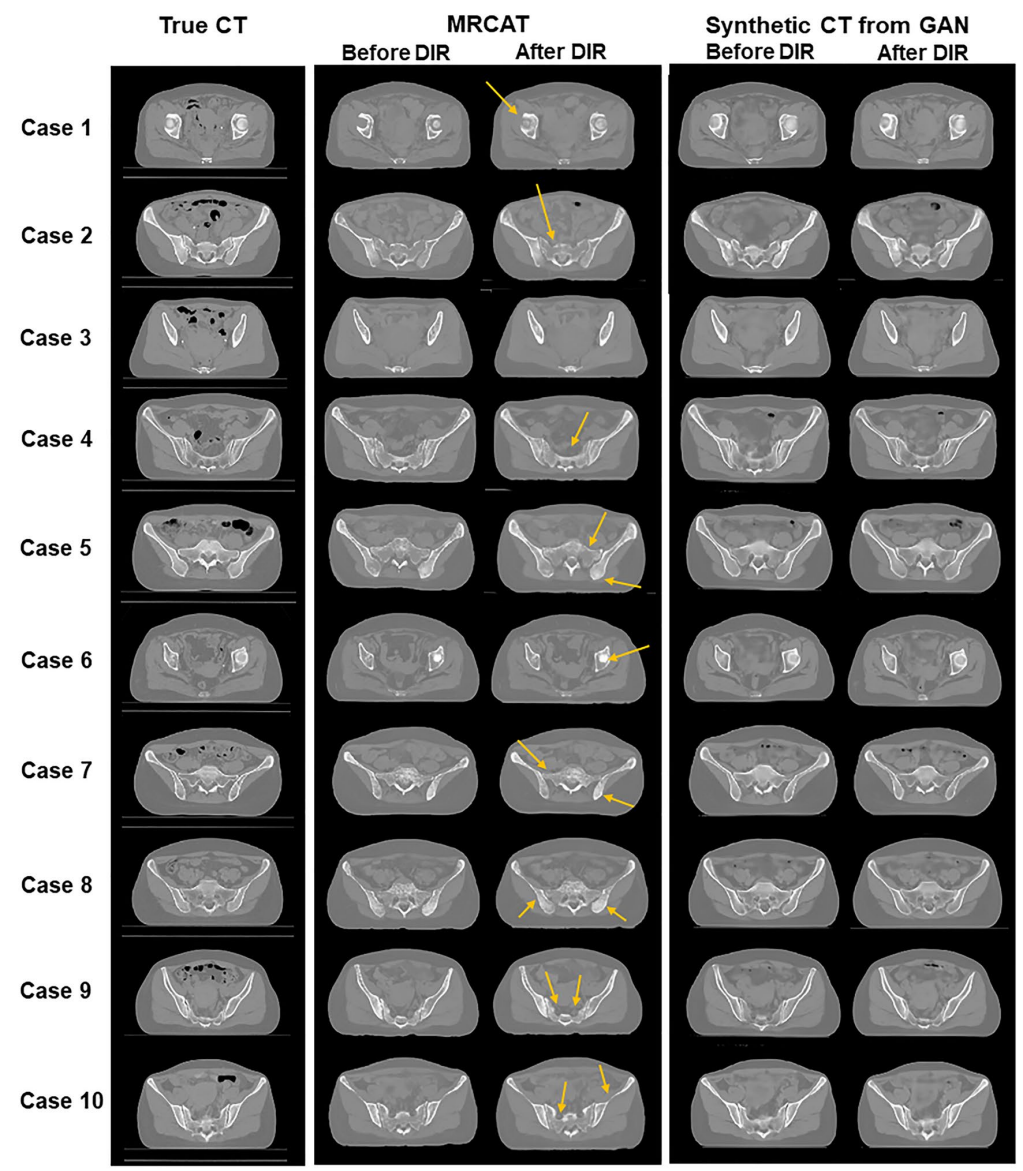
Comparing true CT images (first row) to MRCAT images (second row) and synthetic CT images from deep learning (third row) [− 750 HU ,750 HU].

Figure 1 shows image similarity between true CT images and two types of synthetic CT images: MRCAT and GAN-based CT images. Learning-based GAN produced more qualified synthetic CT images than MRCAT images, as seen in Fig. 1 that included the synthetic images before and after DIR operation. Before applying DIR, the synthetic CT images from GAN had more realistic image texture, which made them look closer to the true CT images than MRCAT images that showed less image contrast and fewer image details. DIR to be conducted for dose calculation in assessment appeared to reduce the difference between two-types of images, while the deep learning yielded more realistic synthetic images. The difference became explicit on the bone anatomy, as indicated by arrows in yellow in Fig. 1. MRCAT frequently under- or over-estimated the CT intensities on the bony structures, while deep learning improved the detailed description on the bony structures.

The enhancement in image similarity throughout deep learning was also found in the quantitative analysis, as listed in Table 1. It turned out that the deep learning-based approach led to more accurate synthetic CT images than MRCAT did. The GAN-based synthetic CT images achieved SSIM of 0.9799, and MAE of 10.97 HU, which were about 1.4% greater in SSIM, and 18 HU lower in MAE than MRCAT images had. The improvement across the 10 testing cases was statistically significant (*p* = 0.00) when we analyzed the results by a paired-samples T-test after passing the normality test in SPSS.

**Table 1:** Image similarity between true CT and two-types of synthetic CT images: MRCAT and GAN-based CT images.

		1	2	3	4	5	6	7	8	9	10	Avg
SSIM	MRCAT	0.9685	0.9611	0.9668	0.9624	0.9634	0.9625	0.9671	0.9709	0.9636	0.9700	0.9656
	Synthetic CT from GAN	0.9811	0.9745	0.9784	0.9795	0.9779	0.9774	0.9799	0.9854	0.9783	0.9866	0.9799
MAE (HU)	MRCAT	39.99	35.33	34.12	34.01	26.76	30.45	22.06	22.66	29.13	21.21	29.57
	Synthetic CT from GAN	10.80	14.38	11.64	10.75	12.31	12.22	9.99	8.47	11.74	8.13	10.97

Besides image similarity, clinical feasibility of two types of synthetic CT images was assessed in dosimetry similarity. The dose was computed by a commercial Monte-Carlo algorithm on the deformed MRCAT and GAN-based synthetic CT images with the SIB-VMAT plan that was optimized with the true CT image for each testing case. Table 2 listed up the numerical results regarding the differences between the dose distributions on true CT and two types of synthetic CT images in GPR at 1% and 1 mm criterion with > 4.5 Gy and > 27 Gy cut-off dose values. MRCAT attained 97.84% passing rate when the region of interest was defined as the imaging voxels that received above 5 Gy, which is 10% of the prescription dose (45 Gy). Contrarily, the synthetic CT produced from deep learning-based approach reached 98.71% passing rate, which enhanced the GPR 0.9% over MRCAT. The difference in GPR between two synthetic CT images ranged from 0.5% on case 6 to 2.1% on case 9. The bigger dose cut-off value (27 Gy, 60% of the prescription dose) was applied in calculating GPR to constrain the region of interest to high dose of radiation. The extent of improvement in dosimetry accuracy achieved by synthetic CT images from deep learning over MRCAT was shown to be greater on high dose region, leading to 5% difference on average, as seen in Table 2. The enhancements were made without any exceptional cases out of the 10 testing datasets, which made the differences statistically significant (*p* = 0.00) from the paired-samples T-test.

**Table 2:** Dosimetry similarity in gamma passing rate (GPR, %) at 1% and 1 mm criterion with 10% (4.5 Gy) and 60% (27 Gy) cut-offs of the prescription dose between dose distributions computed on true CT and two-types of synthetic CT images: MRCAT and GAN-based CT images.

		1	2	3	4	5	6	7	8	9	10	Avg
GPR (1%/1 mm) (> 4.5 Gy)	MRCAT	98.44	97.47	97.44	96.53	98.13	99.05	97.34	99.00	97.53	98.38	97.84
	Synthetic CT from GAN	99.34	98.67	98.16	97.52	98.87	99.53	98.47	99.44	99.64	99.03	98.71
GPR (1%/1 mm) (> 27.0 Gy)	MRCAT	92.20	88.23	90.01	87.22	92.46	95.31	88.09	95.77	87.34	95.55	91.22
	Synthetic CT from GAN	97.04	94.42	93.88	95.37	96.03	98.30	94.44	98.05	98.36	98.05	96.39

**Figure 2:**
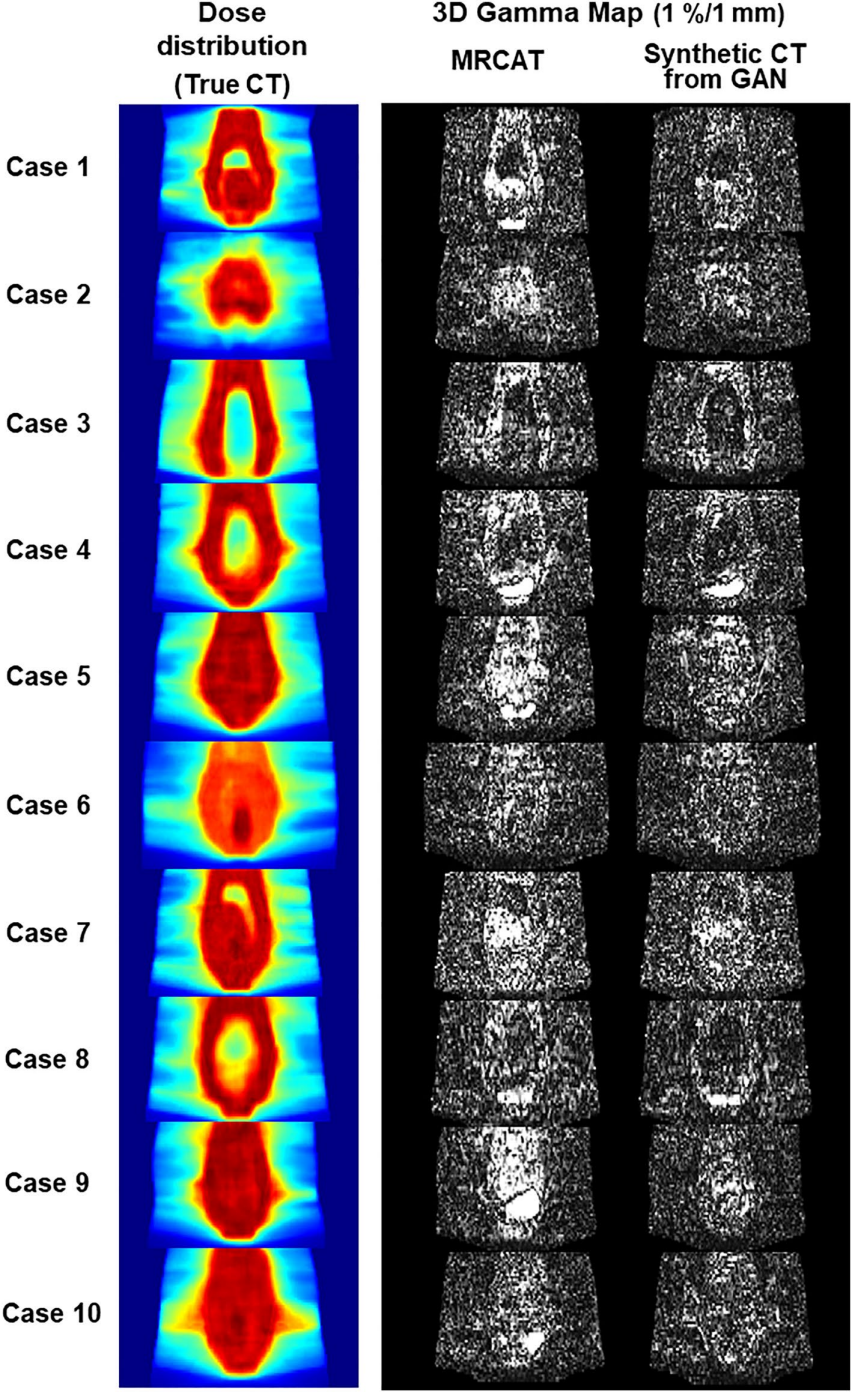
Dose distributions in a coronal plane on 10 testing cases (left column), and gamma maps (1%/1 mm criterion) between dose distributions on true CT and two types of synthetic CT images: MRCAT (middle column) and synthetic CT images from GAN (right column).

Figure 2 visualized (1) dose distribution computed on the true CT images in coronal axis and (2) 3D gamma maps for the MRCAT and GAN-based synthetic CT images for the 10 testing cases at 1% and 1 mm criterion. The gamma maps in Fig. 2 were constrained between 0 and 1, in which the voxels greater than 1 were considered to be unmet to the criterion (1% and 1 mm) required. It highlighted that the deep learning-based synthetic CT images had smaller number of voxels with high intensity than the MRCAT images had. There were smaller number of voxels inside or around the high dose region that were high and bright in the gamma maps produced from the deep learning over the MRCAT images. These results possibly supported our observation above that the accurate intensity prediction of synthetic CT images accomplished by deep learning could have greater impact on the high dose region.

Table 3 listed up the image similarity between MR images and synthetic CT images before applying DIR in SSIM and dice similarity coefficient (DSC) for the body contours delineated on each image. It revealed that the SSIMs between MR and synthetic CT images without DIR behaved similar across the 10 test datasets, leading to the difference of 0.0022 on average. Also, the DSCs for the body contours delineated on MR and two synthetic CT images without DIR were quite close to each other for the respective cases, resulting in the difference of 0.0008 on average. The implication from the results was that the improved performance of the deep learning in generating the synthetic CT images was not attributed to the role of deformable image registration. The difference of performance was most likely to be derived from the synthetic CT generative methods.

**Table 3:** Image similarity in SSIMs and DSCs between T2W MR and two types of synthetic CT images (before applying DIR): MRCAT and GAN-based CT images.

		1	2	3	4	5	6	7	8	9	10	Avg
SSIM	MR vs. MRCAT (without DIR)	0.8124	0.8144	0.8217	0.8184	0.8170	0.8056	0.8082	0.8093	0.8079	0.8103	0.8125
	MR vs. Synthetic CT from GAN (without DIR)	0.8122	0.8159	0.8234	0.8211	0.8199	0.8082	0.8110	0.8119	0.8109	0.8128	0.8147
DSC (Body Contour)	MR vs. MRCAT (without DIR)	0.9922	0.9859	0.9928	0.9894	0.9917	0.9886	0.9904	0.9908	0.9916	0.9922	0.9906
	MR vs. Synthetic CT from GAN (without DIR)	0.9908	0.9833	0.9920	0.9926	0.9928	0.9912	0.9922	0.9925	0.9927	0.9935	0.9914

## Discussion

This study was motivated by synthetic CT generation from T2-weighted MR images throughout a deep learning model, with the aim of demonstrating the clinical feasibility of the synthetic CT images for potential use in radiotherapy. It is well known that several methods have been developed to generate synthetic CT images from MR images, in which the analytical segmentation-based approach was adopted in the commercial MR scanners and simulators known as MRCAT. Since MRCAT has been clinically approved and is currently capable of generating synthetic CT images for certain body sites, the comparison and verification of the synthetic CT images by deep learning against MRCAT would be necessary to demonstrate its clinical feasibility. Thus, we applied a deep learning-based framework for cervical cancer patients, in which the MRCAT pelvic protocol is available. To the best of our knowledge, this study represents the first trial on investigating the clinical feasibility of the deep learning model in the context of synthetic CT generation from MR images, particularly in comparison to commercial MRCAT images.

Several attempts were made in this work to obtain optimal results in the synthetic CT generation and verify the clinical relevance of the framework. Firstly, great attention was given to enhancing the similarity between pairs of MR and CT images. All MR and CT images used in this work were scanned on the same date and under a very similar condition (empty-bladder) for each patient without exception. The MR images were both rigidly and non-rigidly registered to the corresponding CT images. Secondly, the data consistency was also strengthened. As CT images were used to compute the dose distribution, CT imaging data employed for training and testing a network was sourced from a single CT simulator. For MR images, to reduce the deviations in MR image intensity across different patient cases, the Nyul intensity normalization was performed. Thirdly, apart from data pre-processing, we implemented one of the representing generative model, GAN. Though various generative models have been proposed, GAN has been recognized the most successful network architecture and served as a reference to verify the performance of the new deep learning models. Lastly, this study emphasized the dosimetry similarity to verify the clinical availability of the generated synthetic CT images and to compare them to the commercial MRCAT images. For dose calculations on the synthetic CT images, the Monte Carlo algorithm was employed to ensure more accurate quantitative analysis between two types of synthetic images.

When applying the trained network to the 10 clinical cases in the testing phase, it was observed that the synthetic CT images generated by the proposed GAN model outperformed the MRCAT images in both image and dosimetry similarity, compared to the true CT images. The improvements over the MRCAT synthetic images were statistically significant in all categories. The GAN-based synthetic CT images had MAE of 10.97 HU in image similarity, relative to the true CT images, which helped achieve 98.71% and 96.39% passing rate on average in dosimetry similarity of GPR at 1%/1 mm criterion for 10% and 60% dose cut-offs, which were 0.9% and 5.1% greater than MRCAT images attained. The quantitative results showed the advantages of deep learning-based framework over MRCAT that has been clinically used, thereby successfully establishing the clinical feasibility. The improvement in dose calculation accuracy would yield more reliable, secure treatment plan as the treatment planning optimization finds the beam intensity map by minimizing the difference between the computed dose and ideal dose distributions. This work also proved that the enhancement achieved by the deep learning-based approach in generating synthetic CT images was not derived from the registration, including DIR. Even before DIR applied, the DSCs of the body contours of the original T2W MR and resulting synthetic CT image from the deep learning exceeded 99%, which was almost similar to or slightly above those of the MR and MRCAT images.

Such an improvement in generating the synthetic CT images from the deep learning against the MRCAT-based approach would grant a couple of benefits in MR-only radiotherapy. The MRCAT images were obtained from the segmentation-based method, which normally required multi-echo images T1-weighted MR images to be scanned. It took more time to acquire the T2-weighted MR images that are the most widely used for tumor delineation in radiation oncology in addition to the MRCAT procedure. Though the deep learning demands the additional computing system that is devoted to generating the synthetic CT images, it would not intervene in the image acquisition procedure. Also, though having been improved, the segmentation-based approach for the MRCAT still produced unrealistic texture in bony structures, as seen in Fig. 1. Besides, the deep learning-based approach produced slightly more accurate image translation for bladder and gas in bowel. The MC-based dose calculation would have been able to elucidate the dosimetry difference derived from the slightly different image intensities. The difference in dosimetry between the MRCAT and the deep learning might have been due to the degree of prediction accuracy for such structures of the synthetic CT images.

Despite various advantages stated in this work, there are a couple of limitations of the proposed framework. The first limitation was that the deep learning-based workflow for synthetic CT image generation may well demand a site-specific trained model for clinical applications. There must be an additional effort and time required, which has been a common barrier of the deep learning-based frameworks applied for image segmentation and automated treatment planning for radiation therapy. Secondly, as seen in Fig. 1, the predicted synthetic CT images did not fully describe the gas in bowel and bladder although there was a slight improvement compared to the MRCAT images. It is worth noting that recent advancements in generative models, such as the diffusion probabilistic model and vision transformer, hold promise for addressing these imperfections in the prediction of synthetic CT images. The vision transformer was known to be more robust in the image generation by takin more global image information with self-attention operation, and the diffusion probabilistic model that leans toward unsupervised learning would be able to better estimate the gas in small bowel for the cervical cancer patients. Thirdly, the evaluation process emphasized the similarity in image and dosimetry aspects. While enhancing the performance of the synthetic CT image, we would be able to adopt different validating approaches, such as image segmentation^47–49^ and feature extractions^50–52^ for treatment outcome modeling throughout the generated synthetic CT images, compared to those tasks on the real CT images. Finally, the number of patients cases used for training (40) and testing (10) the network, might be considered relatively small. Instead, our primary focus was on maximizing the image similarity and data consistency by strengthening the pre-processing steps, ensuring rigorous verification procedure, and optimizing hyper-parameters on the GAN network architecture. The proposed workflow, as outlined above, achieved superior accuracy in synthetic CT generation, relative to the clinically available MRCAT framework. In the future, incorporating additional options and techniques could further elevate the performance of generating synthetic CT images from MR images.

## Methods

### Patient cohort

All research was performed in accordance with relevant guidelines and regulations. The ethics committee/institutional review board of the Yonsei University Severance Hospital, Korea (4-2022-0311) approved the study protocol, and waived the need for informed patient consent for the retrospective analysis of patient images. The patient cohort for this study consisted of 50 pairs of MR and CT images from cervical cancer patients, which were split into 40 pairs for training, and 10 pairs for testing a proposed deep neural network. CT images were scanned at CT simulator to be used for treatment planning and actual treatment. MR images were T2-wegithed (T2W) MR images that have been widely used to define the target volume for radiotherapy due to its ability to highlight image contrast between normal and tumor tissue. CT images were acquired using a single CT simulator (Canon Aquilion LB, Canon Medical Systems Corporation, Japan), and T2-weighted MR images were obtained from MR Ingenia 3.0 T simulator (Philips Healthcare, Amsterdam, Netherlands). The MR and CT images had different voxel spacing, 1.06 × 1.06 × 3 mm^3^ for MR images and 0.76 × 0.76 × 3 mm^3^ for CT images. To minimize the discrepancy between the different imaging modalities, MR images were scanned followed by CT images on the same day within a few hours. For the same reason, the patients were instructed to make bladder empty during the scanning of both MR and CT images.

The 10 patient cases belonging to the testing phase received the simultaneous integrated boost-based volumetric modulated arc therapy (SIB-VMAT) with two or three arcs for cervical cancer. Among these cases, seven had three target volumes (2.2 Gy, 2 Gy, and 1.8 Gy × 25 fractions), two had two target volumes (2 Gy and 1.8 Gy × 25 fractions), and only one had a single target volume (1.8 Gy × 25 fractions). All test cases in the dataset were provided with MRCAT images generated by mDixon sequence embedded in the MR simulator^15^, in addition to T2-weighted (T2W) MR images. These MRCAT images were used for comparison against the synthetic CT images produced from the proposed deep learning framework.

### Data preprocessing and network training

The pairs of T2W MR and CT images were used to train a deep CNN, where the T2W MR and CT images were defined to be the input and output images. The network was designed to generate the synthetic CT images from the T2W MR images, in which the generated synthetic images were updated by comparing it to the true CT images throughout a loss function.

**Figure 3:**
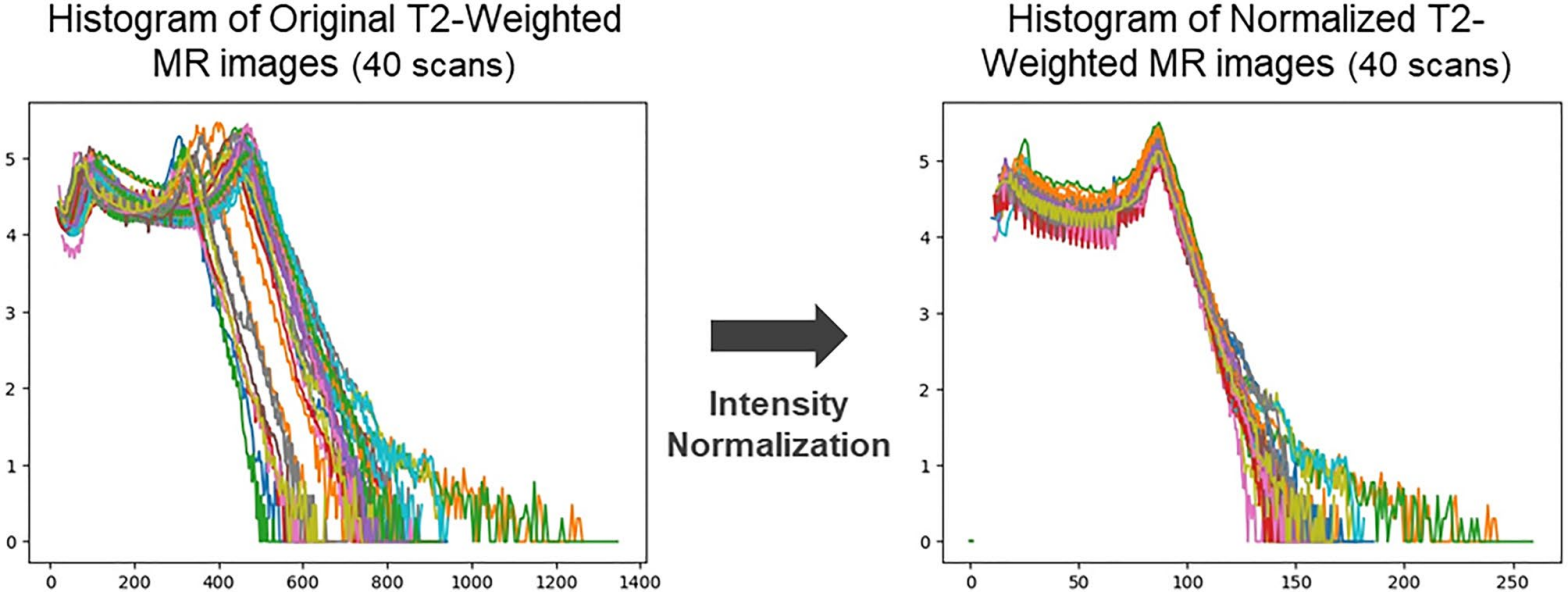
Impact of Nyul image intensity normalization applied to T2-weighted MR images: (Left) Histogram of unnormalized (original) images, (Right) Histogram of normalized images.

**Figure 4:**
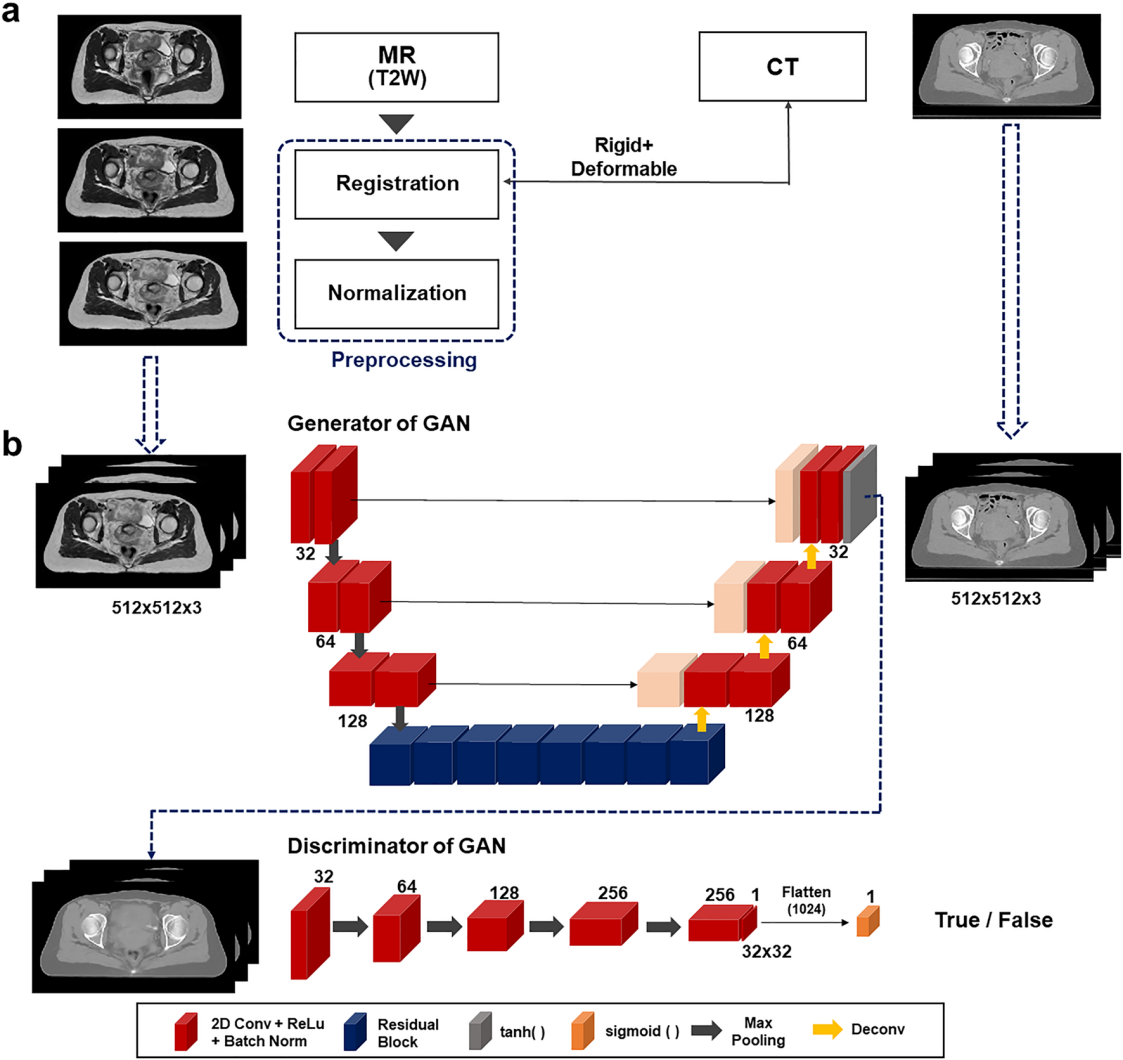
(**a**) Pre-processing MR images before network training: image registration and intensity normalization, (**b**) Network architecture based on GAN for synthetic CT generation from T2-weighted MR images.

This work aimed to reinforce the degree of similarity between input and output of the network, corresponding to the given MR and CT images. To achieve this goal, the MR and CT images were simulated on the same date with bladder empty, as stated above. Also, a couple of pre-processing steps were adopted to further enhance the image similarity. It was found that the intensity distribution of the MR images slightly varied across patient scans despite using the same simulator and pulse sequence. Several studies^53–55^ employed normalization process for the MR images to constrain the variations in MR image intensity. This work adopted a piece-wise linear histogram matching method, called Nyul normalization^56^, which is also a data-driven normalization approach. It was designed to apply a standard histogram to a set of MR images (40 MR scans in this work) for training the network. Each image in the given set was normalized with reference to its maximum and minimum values, which can help define the standard scale landmarks by averaging the image values at 10% interval e.g. 1%, 10%, 20%, …, 90%, and 99%. With this new standard scale landmarks, the image in the set was discretized into different segments depending on the intensity (group 1 is the elements whose image intensities were between 1 and 10%, for instance) and newly normalized in each segment. Figure 3 shows histograms of the MR images before and after applying the intensity normalization, which contributed to enhancing the consistency of intensity distributions across the MR image datasets. As stated in the preceding section, the normalization technique was not used for the CT images, since there were little histogram deviations in CT images across the patients obtained from a single CT simulator. The normalized MR images were rigidly registered to the CT images, such that the registered MR images had the same imaging parameters as the CT images, which can facilitate the evaluation for the generated synthetic CT images. To further refine the registration, and minimize potential anatomical changes between the simulations, the T2W MR images were non-rigidly registered to the CT image for each training case using deformable image registration (DIR). Figure 4a specifies the pre-processing steps applied to this work.

The pre-processed MR images along with the CT images were used as input for training the network. The small datasets with 40 patient scans was not sufficiently large for the 3D-based network training. The 2D-based setting could take a number of pairs of axial MR and CT images for network training, while it might be able to neglect the 3D volumetric changes. Hence,the MR and CT images entering the network were designed to have three slices of the 2D axial images, yielding a matrix shape of 512 × 512 × 3. This pseudo-3D, also known as 2.5D setting, took into account the slices above and below of a specific axial slice during the network training, potentially being able to conduct 3D-like training with insufficient data availability. Predicting synthetic CT image from T2-weighted MR image was performed by generative adversarial network (GAN), as illustrated in Fig. 4b. GAN has been considered one of the most successful network architectures for the image generation, being widely used for a benchmark for the newly developed network architectures. Unlike conventional CNNs with a single generator, GAN had an additional structure called discriminator that compares the real image and predicted image from the generator^28,40,57^. The goal was to achieve a level where the discriminator is hard to distinguish between the real and the generated images. The structural characteristics established adversarial, competitive relationship between the generator and discriminator during the network training that can contribute to helping enhance prediction accuracy. The backbone network for the generator was a conventional U-Net-based architecture with skip connections that help preserve image gradient information in deconvolution process, featuring a residual structure in the bottle-neck. The loss function of the generator was defined as L1-loss, in addition to the adversarial loss. The discriminator had five layered down-sampling convolution blocks with ReLU activations, followed by a sigmoid function for binary classification.

### Evaluation and implementation

**Figure 5:**
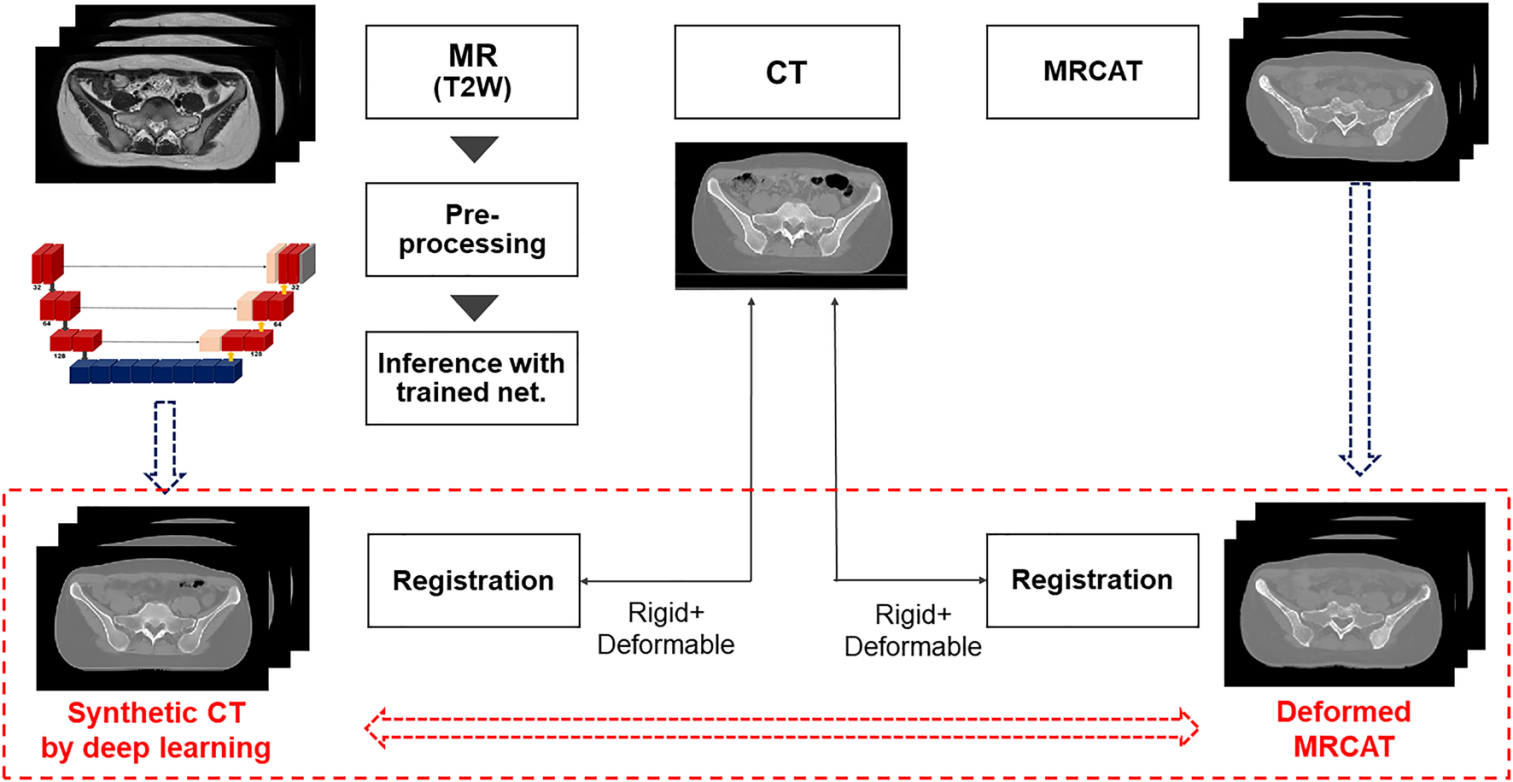
Evaluation of two types of synthetic CT images: MRCAT and GAN-based, where both were re-registered to the true CT images for quantifying image and dosimetry similarity.

For the 10 independent testing cases, MRCAT images were obtained from the mDixon multi-echo sequence along with the T2W MR images in MR simulation. The mDixon multi-echo sequence acquired two T1-weighted MR images, which were decomposed into in-phase (water + fat), water and fat images. The in-phase image was used to extract the bony structure, while the water image corresponded to the soft-tissue for the MRCAT images. The synthetic CT images from the deep learning framework were compared against the MRCAT images using the same criteria. Though the pairs of MR and CT images, including MRCAT images, in the testing datasets were acquired on the same date, it was found that the images were not perfectly well aligned each other. Thus, as described in Fig. 5, DIR following rigid registration was performed on the resulting synthetic CT images after the network inference and on the MRCAT images to the true CT images. This was a necessary process to ensure that the two types of synthetic CT images could be properly compared and assessed. Without this step, it would be challenging to determine whether the improved performance of a specific technique was derived from the accuracy of the density (intensity) prediction from resulting synthetic CT images or the accuracy of image registrations.

**Figure 6:**
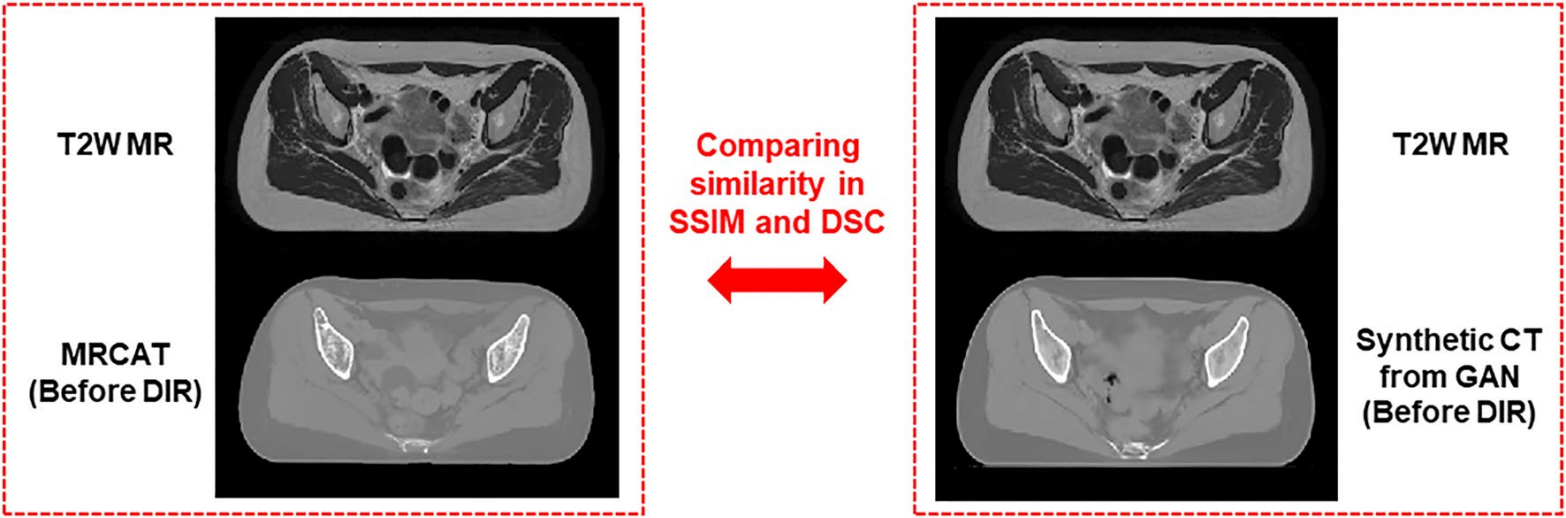
Comparing image similarity between (1) T2W MR and MRCAT (with no DIR), and (2) T2W MR and GAN-based synthetic CT images (with no DIR) to identify that the deformable image registration is independent of superiority of a specific approach against the other in generating the synthetic CT images.

To additionally check the registration effect from the DIR on the evaluation, the image similarity was quantified in SSIM and DSC for T2W MR image and two types of synthetic CT images, as seen in Fig. 6. For DSCs, we delineated the body contours for MR and two synthetic CT images for each test dataset with an aid of a treatment planning system, RayStation 11B (RaySearch Laboratory, Sweden). This was a compelling process to show that the superiority of a specific method for generating the synthetic CT was not originated from the image deformations if the DSCs and SSIMs were similar between MR and two respective synthetic CT images.

The quantitative assessment of the synthetic CT images produced from T2W MR images was performed in terms of both image and dosimetry similarity, relative to the true CT images. The image similarity between true and synthetic CT images was conducted by conventional approaches, which measured the mean-absolute error (MAE) and structural similarity (SSIM) between the two types of synthetic CT images and the true CT images. Dosimetry similarity was compared by analyzing the dose distributions on the true and synthetic CT images generated by the deep learning model and MRCAT. The dose calculation on the true and synthetic CT images was performed on a commercialized TPS, MONACO (Elekta Solutions, Stockholm, Sweden) with X-ray Voxel Monte Carlo (XVMC) dose calculation engine. The same clinical SIB-VMAT plan was applied to three different CT images (true and two types of synthetic CT images) for each test case. The computed dose distributions on the synthetic CT images were compared against the reference dose distribution on the true CT images in gamma passing rate (GPR) at 1% and 1 mm criterion. Typically, GPR is calculated with 10% cut-off value of the prescription dose. However, in this study, GPRs were produced with both 10% and 60% cut-off values to highlight the dosimetry impact of the synthetic CT images in the high-dose region.

This work used Precision treatment planning system from Accuray (Accuray Incorporate, USA) for DIR between multi-modal images (MR and CT images). The mono-modal DIR on the testing phase was performed by an open-source software Plastimatch (http://www.plastimatch.org)^58^ with a three-layered multi-resolution approach. GAN was implemented in TensorFlow 1.14 (http://www.tensorflow.org) and Python 3.6 (http://www.python.org) on a personal workstation with an accelerated GPU (Nvidia GPX Titan X). The network architectures of the generator and discriminator were illustrated in Fig. 4b. The pre-processed CT and MR images entering the network were normalized, so that the intensity of those images ranged from -1 to 1 for the network training. As stated before, the loss function was defined as a summation of L1-loss and adversarial loss, in which the weights for L1- and adversarial losses were defined to be 1 for both. The Adam optimizer was used for training the network with a mini-batch size of 3 by a learning rate of 2 × 10^–4^. The number of epochs was set to be 100. Statistical analysis regarding image and dosimetry similarity between two different types of synthetic CT images was conducted in SPSS (IBM, USA).

## Conclusion

This study has demonstrated the potential power and effectiveness of applying a deep learning-based workflow to generate synthetic CT images from T2-weighted MR images for cervical cancer patients. The deep learning-based synthetic CT images achieved an SSIM of 0.9799 in image similarity and a GPR of 98.71% in dosimetry similarity on average. Notably, these results surpassed the MRCAT images obtained from the MRCAT pelvic protocol that has been approved for clinical use. Conclusively, these findings indicate that the synthetic CT images derived from deep learning-based workflow has accomplished the clinical feasibility, thereby offering promise for integration into radiotherapy.

## Data Availability

The datasets generated during the current study will be available from the corresponding author on reasonable request.
